# Leadership Experiences Amongst Elite Female Rugby Players: A Different Approach to Team Leadership

**DOI:** 10.3390/bs16040606

**Published:** 2026-04-19

**Authors:** Stewart Cotterill, Richard Cheetham

**Affiliations:** 1 School of Health and Rehabilitation Sciences, Health Sciences University, Bournemouth BH5 2DF, UK; 2 School of Sport, Allied Health and Social Work, University of Winchester, Winchester SO22 4NR, UK

**Keywords:** empathy, authenticity, empowerment, gender, leadership

## Abstract

Leadership, and athlete leadership in particular, has been reported to be an important factor impacting upon team performance. However, while there is significant evidence supporting the importance of athlete leadership for teams, there is very little research exploring the leadership experiences and needs of female sports teams. As a result, the aim of this study was to explore the leadership experiences of the captains of professional women’s rugby teams. Participants included eight professional women’s rugby captains, recruited through personal contact. Data were analyzed adopting an interpretative phenomenological analysis (IPA) approach, resulting in the emergence of 10 superordinate themes including: factors influencing success, challenges, amateur level, leader characteristics, role models, aspects of the role, types of captains, leading by example, selection, and women’s game. Data suggests that empathy, empowerment, collaboration and shared/devolved leadership are crucial components of leadership for elite women’s rugby teams.

## 1. Introduction

Effective leadership has been consistently highlighted as an important factor influencing the success of sports teams (Mach et al., 2022). In particular, the importance of leadership within these sports teams, provided by the athletes themselves (athlete leadership) has been highlighted to be crucial for team success and additionally for a range of associated positive team outcomes (Cotterill & Fransen, 2016). The increased focus on athlete leadership in recent years has served to deepen our understanding of leadership functioning within sports teams, and crucially has underpinned the creation of specific development programmes to further enhance leadership provision and support within these teams (Cotterill et al., 2022). However, while this increased focus on athlete leadership is welcomed there has, to date, been limited exploration of athlete leadership and leadership development within women’s sport, and specifically in elite and professional women’s sport.

Athlete leadership has been described to be “an athlete occupying a formal or informal role within a team who influences a group of team members to achieve a common goal” (Loughead et al., 2006, p. 144). More recent categorizations of leadership within sports teams have distinguished between four different leadership roles that athletes can occupy in the team: task, social, external, and motivational (Fransen et al., 2014). Specifically, the task leader gives teammates tactical advice and adjusts this when necessary; the *motivational leader* encourages teammates to perform at their best; the social leader develops a positive team atmosphere; and the external leader represents the team towards external parties such as club management, media, or sponsors (Fransen et al., 2014). It has long been assumed that the captain is a key in meeting some of these different leadership needs within the team (Cotterill & Fransen, 2016). Though many of these athlete leadership roles are emergent within the group, at the professional sport level, the position of captain is often appointed by the club, and often specifically by the coach (Cotterill, 2013). Research within professional rugby union (Cotterill & Cheetham, 2017; Cotterill et al., 2019) has highlighted the important role that the captain plays in the sport, and the leadership dynamic these captains develop with the coach. However, what has also emerged is the lack of real clarity regarding the requirements of the role and how effective leaders of the future can be developed.

There has been an increasing number of research studies in recent years exploring athlete leadership in a variety of domains and contexts (Cotterill & Fransen, 2016). However, while this has seen a significant expansion of knowledge and understanding relating to athlete leadership much of this research has been with male populations, with recommendations applied and generalized across both male and female sports. More recently a small number of studies have sought to explore different leadership experiences across both male and female teams. For example, Mertens et al. (2021b) in their intervention study applying the 5Rs leadership programme reported a consistent impact on leadership development across genders. While this reported outcome is encouraging other recent studies suggest that gender differences are starting to emerge. In a study exploring leadership develop in student athletes in the USA Ohlson et al. (2022) reported significant positive outcomes from engagement with the programme for both male and female athlete leaders. However, while this was true for both males and females the authors also reported that the greatest positive effects were amongst female participants highlighting “the importance of customized gender-specific leadership development” (p. 85). The positive impact of female-specific leadership development programmes has also been reported in a study of a female lacrosse team in the USA (Gellock et al., 2019); and across basketball, lacrosse and soccer in Japan (Takamatsu & Yamakita, 2022). While the direction of this research is encouraging there are still relatively few studies focused specifically on leadership in women’s sport.

There are also still relatively few studies explicitly focused on leadership experiences at the highest level in sport. Indeed, Cotterill and Cheetham (2017) in their study of leadership experiences in elite men’s rugby union highlighted a lack of research that explores the leadership experiences of athletes at this elite level in male sport. This observation is even more apparent for elite women’s sport. Recently Andrews et al. (2022) explored differences perceptions in the role of athlete leaders in their study of elite women’s cricket. The study specifically explored the differing perceptions between female athlete leaders and the male coach in an Australian elite women’s cricket team. The authors concluded that further research exploring gender differences in leadership perceptions and approaches was required. In addition, Mueller et al. (2022) conducted a social network study of the women’s USA rugby union 7s team. The authors highlighted the important role the emergent leaders from the team had in supporting those athletes in formal leadership positions such as captain.

While there has been a significant expansion of contemporary knowledge around the role, function, and development of athlete leaders in sport much of this research has focused on male athletes and male sport at the sub-elite level. With this work often serving as the foundation for approaches to leadership and leadership development across sport. This knowledge has also been applied to women’s sport even though there is a lack of clarity regarding whether the perceptions of the role and function of athlete leaders in women’s sport is the same as in male sport. In addition, emerging research suggests that the best approaches to the development of athlete leaders may differ based upon the gender of the group.

As a result, based upon the limited research exploring the perceptions of elite athlete leaders in women’s sport, and specifically in the sport of rugby union, the aim of this study was to better understand the nature of leadership roles within women’s rugby at the elite level. Seeking to understand the perceptions and experiences of captains in exploring the role of the leader, the selection process, and the relationship between the captain, the coach, and the team.

## 2. Method

### 2.1. Design

Similar to the approach adopted by both Cotterill and Cheetham (2017) and Cotterill et al. (2019) in professional rugby union the current study adopted an interpretative phenomenological analysis (IPA approach). This approach has been increasingly applied to the field of sport psychology in recent years (DeWolfe & Dithurbide, 2022). IPA as an approach is best suited to forms of data collection that invite participants to articulate stories, thoughts, and feelings about their experiences of specific phenomena (J. A. Smith, 2004). The approach offers methodological flexibility allowing for a more individual application of the relevant steps in the research process (Cope, 2011). The approach offers a detailed analysis of the personal accounts of participants followed by a presentation and discussion of the generic experiential themes that is typically paired with the researcher’s own interpretation.

The research project adhered to University of Winchester’s ethical guidelines and obtained ethical approval from the Faculty of Business, Law and Sport Research Ethics Panel (BLS/18/19). Specific attention was dedicated to issues of confidentiality, anonymity, and informed consent throughout the research process. Participants were provided with a consent form that included an information sheet about the project and were given the opportunity to ask questions before giving their consent. The digital recordings and transcripts were kept in a secure, password-protected area, accessible only to the research team. Moreover, the transcripts were anonymized, and any details that could identify the participants were removed. Confidentiality and anonymity were maintained by using pseudonyms when reporting the findings, including the writing of this article.

### 2.2. Participants

The current study adhered to J. A. Smith and Osborn’s (2015) guidance for IPA studies and the purposeful selection of a homogenous sample. Participants were selected based upon their experience as elite (professional) female rugby players. Specifically, participants were recruited through personal contact, who were known to the authors from professional rugby clubs in the English Premiership Women’s Rugby (elite national professional league), based in the UK. There were eight female participants in total, with an average age of 30.67 years old (ranging between 27 to 34 years old) who had been playing rugby on average for 11.33 years in professional/semi-professional rugby (ranging between 6 and 16 years of experience), who had been club captains for an average of 3.16 years as captain (ranging from 2 to 4 years).

### 2.3. Procedure

The participants were interviewed to gain an insight into their experiences of leadership as captains of professional women’s rugby teams. Semi-structured interviews were used to explore the participant’s personal narratives and experiences. The use of semi-structured interviews has been outlined to be a core data collection tool for IPA studies (J. A. Smith & Osborn, 2015). A specific interview schedule was developed for this study. This approach to data collection is consistent with the phenomenological approach where the participants are considered the “experts” and it is the meanings that participants associate with their experiences that is of interest to the researcher (J. A. Smith, 1996). The specific process for developing the interview schedule adhered to a four-step approach developed by J. A. Smith and Osborn (2015) for IPA studies. This approach suggested that the researchers: (a) think about a broad range of issues; (b) put these topics in the most appropriate sequence; (c) think of appropriate questions relating to these areas; (d) and think about possible probes and prompts. Examples of interview questions that formed part of the interview schedule included: “Tell me about your role as a captain, and the key aspects of this role?”; “What are the key lessons you have learnt, or knowledge you would pass on to an aspiring captain?” All interviews were recorded using a digital data recorder and transcribed verbatim to produce an accurate record of the conversations that took place. The interviews lasted between 45 and 80 min.

### 2.4. Data Analysis

The data were analyzed using Interpretative Phenomenological analysis (IPA). Through this process the researchers engaged in an “interpretative relationship with the transcript” (J. A. Smith & Osborn, 2015, p. 64). All transcripts were read several times so the researchers could become familiar with each participant’s account. Initial notes were made in the margin of the transcripts annotating anything identified as interesting or significant. As this process continued emerging theme titles were also annotated on the transcripts. These initial notes were then transformed into concise phrases capturing the qualities of the points annotated. The next step involved the researchers’ making connections between the emergent themes and researcher interpretations (J. A. Smith & Osborn, 2015). As these connections were made a clustering of themes emerged. Checks were then made with the original transcripts to make sure connections still worked with the primary source materials. This step then led to the development of a coherent table of themes. Once the transcripts had been analyzed by this interpretative process a final table of super-ordinate themes was constructed.

### 2.5. Quality of the Qualitative Data

A non-foundational approach to judging the quality of qualitative enquiry was adopted in the current study (B. Smith & Caddick, 2012). The specific criteria for judging the quality of this research included: the contribution it makes to the field, its coherence, sincerity, resonance, and credibility (Tracy, 2010). A key aim of this study was to co-construct knowledge that contributes to the understanding of the players’ perspective on the nature of leadership and captaincy within the team, and to report substantive findings. This substantive reporting of the findings was achieved by using detailed quotes from several specific participants when creating the results section of this manuscript. The coherence of the findings in this study was achieved via discussions with a critical friend (Didymus, 2017), who was utilized to discuss matters such as sampling and data analysis. In this context a critical friend was used to provide an objective voice in the design and implementation of the study. In terms of sincerity and the truthfulness of the data, it appears that rapport was effectively gained because participants spoke openly and fully about their experiences. Evidence of this included the length of the interviews, and those participants mentioned players by name, thus suggesting that they trusted the researchers not to disclose any such confidential information about their role working in an elite and public environment. Regarding resonance, the core aim was to produce findings that are valuable in professional rugby contexts (Tracy, 2010). The credibility of the data was enhanced by spending further time with the participants before commencing the interviews, by sharing each player’s interview transcription back with that individual to encourage reflection and dialogue about the data that had been deemed most pertinent, through maintaining a reflexive journal and an audit trail of the research, and by having a critical friend to scrutinize and discuss pertinent matters. The first author also discussed their interpretations of the data with the second author who served as a critical friend for this process (B. Smith & McGannon, 2018). This critical friend process was utilized to further enhance the rigor of the study by developing themes that were supported by the participant’s accounts and were theoretically sound (B. Smith & McGannon, 2018).

## 3. Results

The IPA of these data highlighted ten super-ordinate themes as well as 51 sub-ordinate themes. These ten super-ordinate themes are as follows: factors influencing success, challenges, leader characteristics, role models, aspects of the role, types of captains, leading by example, selection, the women’s game, and advice to new captains form the basis of the results section, with each super-ordinate theme forming a subsection of the overall Section 3 of this manuscript. All super and sub-ordinate themes are outlined in Table 1.

### 3.1. Factors Influencing Success

An important factor highlighted by participants in the current study as impacting upon their ultimate success as a team captain was the authenticity of the approach they adopted, and the congruence of this approach with them individually. Illustrating this point participant one reflected that:

*I sort of give them the advice that I think I would have liked to have been given myself, which is, it is a leadership journey, you will find a style that will work for you, but it’s important that you put your stamp on it; yes, it has to work for the team, but at the end of the day you have to go home and you have to sort of sit with yourself and be happy that you’re playing and you’re leading in a style that is true to yourself, so, don’t be afraid to put your stamp on it.*

A perspective supported by participant four who stated that:

*you’re the captain now, you’ve got to lead in your own way. Because I think when I was captain, I was trying to be XXXX, or trying to be XXXX, when I should have just been trying to be myself. I think I sort of realized that you have to, it takes a while, but you have to learn your own captaincy style and don’t try to be someone you’re not, that you need sort of, it’s difficult actually, because you have to strike the balance between being a friend and being a captain.*

Another factor highlighted as crucial by participants in the current study was the importance of building relationships with the rest of the members of the team. For example, participant five stated:

*Also, making sure you have good relationships with everybody on your team. Naturally you know friendships you build more readily than other relationships, but you know there’s fifteen on a pitch at a time, so, making sure you have a good relationship with all of them, making sure that they feel like they can come to you.*

A view also reinforced by participant three who reflected:

*You know that some players don’t need as much support maybe as others, and ensuring those players who need the support, or you know these are the rules in this instance, and that penalty was given away for this reason that maybe you didn’t know, and that’s okay, that kind of thing, so, just being a good communicator with everybody. But I think as much happens off the pitch as on, really.*

A further characteristic highlighted as being important for effective leadership in the current study was empathy. In considering this view participant one reflected that “*I remember just being thankful for the opportunity to just sort of pull her aside and go, I can see the red mist descending, let’s just try and just stop it there*”. The view that empathy was crucial in the role was also advocated by participant three who shared that:

*If you think about leadership qualities and the factors that you need to have, relationships and that empathy with players is key. Probably even over performance on the pitch probably, or at least level with that anyway. I’d say your work rate and your accountability on the pitch is up there as well, but yeah, I just think relationships, because you’re going to get the best out of your players if you’ve got a good relationship with them.*

### 3.2. Challenges

One of the challenges outlined by participants relating to the role of captain was the importance of being able to make effective decisions on the pitch and not getting too caught up in the game. Participant five highlighted that:

*I think it was that responsibility of making decision on the pitch, and kind of is it the right one [decision] or not, you know penalty decisions. And also, about things like discipline, really making sure that you are leading by example, you play at your hardest, you’re turning up fully switched on and prepared. The onus is on you to kind of, you decide what everyone has to follow, so you can’t be, you know mouthing off to the referee or anything like that.*

Participants also highlighted the challenge of what you should be doing and how you should be completing the role. For example, participant seven reflected that:

*I can’t think of a time when someone maybe gave me specific advice about being a captain. It’s more that I had watched and absorbed from people who had come before. And like we discussed earlier, good role models or captains that I’ve had, and good coaches, who kind of maybe have given advice that I can’t think of specifically, but yeah, I think it’s that role-modelling. People didn’t necessarily come to me and go, I think you should do it like this, or things like that.*

This idea of not knowing what the role entailed, or ‘being thrown in at the deep end’ was also highlighted by participant five:

*I was a bit thrown in at the deep end sometimes, but then at the same time, I’d been involved in rugby since I was quite young, so, it did feel reasonably natural, and I’ve been around a lot of role models, so it wasn’t too bad, but yeah, I suppose with most other things, like jobs, you would get more help I suppose, or preparation.*

There were also challenges highlighted relating to the impact that being captain had upon the participants themselves. One such example was presented by participant three who outlined the stressful impact of the role:

*When I first did it as a captain, my worry was you know ‘oh, people are looking to me, people are going to blame me if selection is the way it is’, and not that we have the final say, but obviously as a captain you get involved, you have chats to coaches, you know you can give them the insight that they need from the players, and obviously the coach makes the final call, and the selection committee. But I feel that was a pressure, you know not to think that I wouldn’t ever make the right decision on the right grounds, but I think you know people are looking to you, and sort of you know got their world in your hands,*

Indeed, the added stress and pressure associated with the role was suggested to have significant consequences. A fact outlined by participant four who shared that:

*I was looking at that added pressure of me trying to lead by example, that led to me getting injured, because I tore my meniscus, which I mean it’s not going to, I mean I didn’t intentionally tear it, I tore it in a bad tackle; someone tackled me from the side, which my knee went a rally awkward way, but I think I wanted to stay on and lead by example, that I definitely made it a hell of a lot worse, and I didn’t go and get it sorted out when I should have done, which led to me basically being injured for the rest of the season.*

### 3.3. Leader Characteristics

Several of the participants in the current study discussed the characteristics that they felt made an effective leader. Participant one highlighted that:

*It’s normally been somebody who is not only a good player, but has good rapport with the team, you know they’re inclusive, yeah, inclusive and kind I think would be it, and a good communicator, is the definite one, so, you know being clear, but having that good vision.*

In reflecting on the traits of the effective leaders who had captained sides participants had played in, participant six stated “I probably had captains who I thought yeah, I want to do that, and they were inspiring as players, and it made me kind of want to play like them, and yeah, captain like them as well”. In reflecting on what they thought made them a good captain participant two also reflected that:

*I’m not sort of like assertive, I’m more of a hard worker, like a lead by example person and I play well because of the people around me. I think maybe I hadn’t thought of it [captaincy] as an option, but I hadn’t really thought of it before then as something natural for me to be the captain. I am quite reluctant to like step up and lead, but I can do it like if I’m given that role.*

The importance of being able to empower individuals within the team was also suggested to be important. Participant one stated that:

*I think it’s being a captain in the sense this is a group of individuals and yes we come together and we’ve got to play as a team, but each individual has to feel comfortable enough to express themselves in order for the team to work, so, I don’t think be too restrictive would probably be the key, the key takeaway for me.*

### 3.4. Role Models

A very strong theme emerging from the current study was the impact that good captaincy role models had on the participant’s approach to captaincy. For example, participant two reflected that:

*The leaders that I’ve played under as captains, probably the ones that I’ve enjoyed playing under the most are not necessarily the best players on the team, they’re kind of probably more sort of humble and hard workers I guess like me, and I think that’s why I like them people like people like themselves.*

A view also supported by participant seven:

*I’ll talk about XXXX, who is a XXXX captain at the moment, I really liked playing under her, because she’s actually nice. I think, it’s the most successful woman’s club in the UK, and potentially the World, but sometimes it’s not the most positive atmosphere, like when sometimes there are little toxic aspects of it, so, I mean it tends to be people that are involved in the England squad. Whereas XXXX was never like that. Very sort of just friendly and interested in like everyone doing the best that they can.*

In addition, participant two further reflected that:

*I kind of appreciated that the coaches chose her, because it means that people like that are kind of valued, and I see myself as a bit like that, in terms I’m not like a loud person that says a lot of stuff, or like not very overbearing, so, that means, it shows me that sort of player, that sort of person is valued very much in the team.*

Finally, in supporting the same point participant three shared:

*The way she played and the way she nurtured and had empathy with how players needed to be treated was quite obvious, back then even to a nineteen-year-old, I really felt she was, you know she sort of knew how I felt even then and everything, being against all these like England players that I’d never you know experienced before, and she really took me under her wing.*

### 3.5. Aspects of the Role

An important aspect of the role highlighted by the participants in the study related to the relationship with the coach. Importantly, participants cited the role of bridging the divide between the team and the coach. For example, participant five highlighted that “*the other thing I think is the captain is that link between the coaches and players, so, having kind of good links with coaches, kind of good relations with them is important*”.

Indeed, trying to facilitate a more effective relationship between the coach and the team was also highlighted in the study. In illustrating this point participant one reflected that:

*And so, when I took on the role of captain, there was a couple of murmurs of discontent about the coach, and you could sort of see the same thing happening again and bubbling up to the surface, so, I attempted to sort of nip it in the bud, and I went to chat to the players and talk about why they weren’t happy with the coach. And then I went to chat to the coach, and go, she isn’t happy, but I’ve seen the pattern before, so, we need to deal with it and we need to look at how can we improve training or how can we improve your communication on match days, but I’m also going to look at it, I sort of to started to view it as a bit of a phenomenon that happened in women’s rugby and the reasons why that was, so, we started to talk about more of the culture of rugby. I did almost feel like I was this messenger between coach and players.*

However, at times the captain’s standing in the captain-coach relationship was questioned. A point highlighted by participant one who shared:

*But I did feel like the messenger, like I was sort of communicating the coach’s message to the players, and then vice-versa, particularly because there was sort of a smidgen of hostility at that time.*

The all-encompassing nature of the role was also a factor highlighted by participants, such as participant five who reflected that:

*you’re not just the captain for eighty minutes on the day, you are always the captain, you know outside of training, when you arrive, when you are in the car park, people know that’s who you are and that you’re being watched. Everything you do is being taken in from that perspective, so, you are always leading, so, whether you’d be chatting to absolutely everybody you see, making sure, you know the staff behind the bar, that you kind of, yeah, that you’re always thinking about that and that all goes into how you play on the day and how you lead on the pitch.*

There were also other aspects of this leadership role mentioned by participants such as providing social leader, such as that reflected on by participant three:

*Make sure you have social time outside of playing, and that everyone is kind of, that’s where you see different personalities coming out and real bonding happening, so, whether that’s kind of, at Uni sometimes it’s kind of going out and things like that, but also having team meals. Because we’d always used to have a team meal before our games, like breakfast, so that you’re having time where you’re not fully charged in a training situation, you just know each other off the pitch.*

Another aspect of the role highlighted as being important was the requirement to motivate members of the team. For example, participant two highlighted that she had to be:

*Motivating, and trying to get the best out of people around you on the team, but like setting an example of like fighting for the result for the team, not actually physically fighting like starting a fight, it was like battling in the game, so, like showing through effort, like set an example that way.*

A point further reinforced by participant three:

*It was really tight, and I just had to, it was more of like a motivational job, we were losing by a try, and we needed a try to win, and a conversion, so, it was more like I was just trying to slap everyone on the back and get them onto their feet, because everyone was knackered, sort of you know at the end of the game, and it was like we’ve got to dig in this extra bit, if we can dig in now we can this win.*

### 3.6. Types of Captains

It was interesting that participants in the current study while appointed to the role of captain, felt that leadership was a group effort. A point highlighted by participant seven who reflected that:

*I think it’s being a captain in the sense this is a group of individuals and yes we come together and we’ve got to play as a team, but each individual has to feel comfortable enough to express themselves, to help lead the team in order for the team to work, so, I don’t think it’s about me leading it is about us leading it.*

A perspective also supported by participant three who said that:

*For me, I think leadership in the team is a group effort, we all support each other to take the team forward, yes I am the captain but it takes a group of us to really make it happen, you know it is a group effort!*

The participants also suggested that being nurturing was an important aspect of the role. This point was presented by participant one who shared that:

*In the clubs in the South-East, and particularly my university one, the captains tended to favour the more nurturing side. And we had a range of different roles; so, in the captaincy we had some captains who were ‘mums’ and they sort of viewed it as the second family and very much sort of adapted their ‘mum’ role, and just sort of extended it to the rugby family.*

This gendered perspective was also highlighted by participant four who reflected that:

*I don’t want to be really stereotypical, but obviously, women are used to doing a lot of those similar roles around the home, particularly if they’re mums, so, I think it became natural, a natural environment for them to do that. And I think it was almost that was their comfort zone off the pitch, so, it was where they felt most confident.*

### 3.7. Leading by Example

Another important factor discussed by the participants in this study was the requirement for captains in this environment to lead by example. For example, participant five highlighted:

*I think particularly at XXXX, I was playing at quite a high level, so it’s that leading by example on and off the pitch, in terms of like training as hard as you can, you know putting a hundred percent in at all sessions, always being at all of the sessions, commitment I think would be the word.*

A perspective echoed by participant three who mentioned that:

*you know that leading by example is absolutely key, because whatever you do as a captain, you wouldn’t, well you wouldn’t expect anyone on your team to do what you wouldn’t be expecting to do, so if you wouldn’t expect it of yourself, then I wouldn’t expect it of anyone else. Know.*

### 3.8. Selection (To Be Captain)

There were also some interesting insights offered by participants in terms of how captains, and they in particular were selected. Being seen as capable and competent was suggested by participants to be important. For example, participant two shared that:

*I think in women’s rugby, I don’t know if it’s different for men’s, but even just understanding what other people in the team think of you as a captain or want from you as a captain, or think you can bring, as a captain, I know I can say I can bring this, but it’s, quite a lot of time it’s really you don’t realise what other people think of you and that I think, I don’t want to over-generalize, but I think women tend to be a bit more concerned by that.*

There was also some discussion among participants about how to develop and encourage the captains of the future. Participant two shared that:

*We start to think who would be good and can we sort of encourage them to run for captain, and I guess at that point you could start to influence them by preparing them and giving them advice. So, for me, I’m not sure, that a course would have been that helpful, other than perhaps giving me confidence to have got that kind of backing.*

### 3.9. The Women’s Game

A very interesting and important part of the discussions that took place with participants related to the nature of women’s rugby. For example, participant eight reflected that:

*I think Women’s rugby is like such a community. I don’t know what it’s like with Men’s rugby, I’m sure it’s the same, but with Women’s rugby it’s really a community, it’s often the people that are your best friends, and so, it’s not just what you do on a Sunday, it’s all of the time, yeah.*

There were also reflections by participants on the perceived gender inequalities that exist in several of the clubs that participants had experience with. For example, participant four shared that:

*I definitely needed help from someone else, which we just didn’t have, which was really quite sad, because our club, had almost a hundred girls at one point, and the boys club only had twenty more people than we did, and they had three coaches, twice the amount of funding and they were in a lower league, and we had none of that because, well the only reason that we didn’t get it was because we were girls, because they didn’t want to give us any funding.*

The view that there were inequalities between male and female teams in rugby was also highlighted in relation to the expectations of the coaches in terms of the leadership role that captains fulfilled in the team. An example of this perspective was provided by participant 4 who reflected:

*It sometimes feels like they [the coach] is so used to working with men that they seem to expect exactly the same things from their leaders that they do for the men’s team … being aggressive and in your face, not sure it is the same thing for us!*

This view suggests issues in terms of embedded masculinity and expectations transferred from male rugby in the expectations of the role of captain in women’s rugby.

### 3.10. Advice to New Captains

The participants in the current study had several key points of advice for future captains either in their team, or in women’s rugby more broadly. This included participant 1 who reflected that:

*Hindsight is a wonderful thing you know and I’ve learnt more about leadership styles since then, but I was quite naïve that I didn’t have the confidence to put my own stamp on the role, but I don’t think that was because at the time anybody communicated about how I might take the role and adapt it for myself. So it is important for future leaders to feel comfortable developing and nurturing their own style.*

Building on this point participant 3 also reflected that:

*I would give them the advice that I think I would have liked to have been given myself, which is, it is a leadership journey, you will find a style that will work for you, but it’s important that you put your stamp on it; yes, it has to work for the team, but at the end of the day you have to go home and you have to sort of sit with yourself and be happy that you’re playing and you’re leading in a style that is true to yourself, so, don’t be afraid to put your stamp on it!*

These views highlight a desire by participants in the current study to help support future captains and to ensure they do not have to face the same challenges and are ultimately better supported.

## 4. Discussion

The aim of this study was to better understand the nature of leadership roles within women’s rugby at the elite level. Specifically, to understand the perceptions and experiences of captains relating to the role of the captain, the selection process, and the relationship between the captain, the coach, and the team. From the data and themes presented in the previous section several interesting factors emerged that warrant further discussion. These include: the importance of authentic leadership; the crucial nature of relationships, rapport with team members, and empathy; the variety in the challenges associated with the role; the importance of the relationship with the coach for the role of captain; the importance of being nurturing; differences between male and female versions of rugby; and the gender inequalities that exist in the game. Each of these key findings from the current study will be explored in more detail.

The importance of being an authentic leader was highlighted by several participants in the current study. According to Bill George (cited in Watts, 2015) authentic leaders are “people committed to meeting the needs of the interest groups they serve, [whilst] displaying values and self-discipline that inspires others” (p. 1). Building upon this point Walumbwa et al. (2008) suggested that authentic leaders “draws upon and promotes positive psychological capabilities [e.g., confidence, hope, optimism, and resilience] and a positive ethical climate, to foster greater self-awareness, an internalized moral perspective, balanced processing of information, and relational transparency on the part of leaders…fostering positive self-development” (p. 94). Indeed, authentic leaders have previously been suggested to minimize their own personal goals and focus on understanding and supporting those they serve (Howell, 1988), an approach that was highlighted by participants in this study. Additionally, leaders who develop authentic leadership skills have been reported to be perceived as more competent by team members (Garcia et al., 2021).

Developing rapport with team members was also suggested to be important in the current study. The importance of rapport has been implied in some previous studies but has not been reported as explicitly in other athlete leadership studies. This lack of explicit reporting of rapport contrasts with research focused on coach leadership in sports teams where the relationships that exist between coaches and athletes and the building of rapport have been highlighted to be crucial (e.g., Bennie & O’Connor, 2012; Lopez de Subijana et al., 2021).

Participants in this study also highlighted the importance of knowing what the role of captain was, and what is required. This might seem an obvious requirement to undertake the role, but previously studies have shown that the clarity regarding what the expectations of the role are can often be lacking (Bray & Brawley, 2002). This is an area sport still needs to improve as you would not expect to start a leadership role in a work environment without having clarity on what is required and how success will be judged. The importance of role clarity for captains has previously been highlighted in professional rugby union (Cotterill et al., 2019), though the authors also highlighted clarity on the requirements of the role is often lacking. Lack of clarity relating to the role of the captain has also been reported to be a stressor for professional sports captains (M. J. Smith et al., 2017).

The relationship between the captain and the coach was highlighted as another important aspect of the role by participants in the current study. This factor has previously been highlighted in other studies focused on athlete leadership (Cotterill et al., 2019), and specifically in the sport of rugby union (Cotterill & Cheetham, 2017).

A strong message emerging from this study was the importance of the captain being nurturing. This is not a factor that has been particularly highlighted in previous research (Cotterill & Fransen, 2016) but was a theme that was mentioned by multiple participants in the current study. In this context being nurturing can be seen as “helping a plan or a person to develop and be successful” (Wilson & Woodhouse, 2008). A concept that is similar to empowerment, which is described to be “an intentional ongoing process centered in the local community, involving mutual respect, critical reflection, caring, and group participation, through which people lacking an equal share of valued resources gain greater access to and control over those resources” (Cornell Empowerment Group, 1989). Indeed, leaders of sports teams who adopt an empowering approach have been reported to be better leaders (Fransen et al., 2020).

A strong feeling presented by participants in the current study related to perceived differences between the female and male games of rugby union. This fact particularly related to the environments that existed for the different male and female teams. Unsurprisingly, differences between male and female sport environments have previously been highlighted in the research (Warner & Dixon, 2013) and explicitly in female rugby union. For example, Cotterill et al. (2020) highlighted that there is a greater degree of emotional contagion for female players when compared with their male counterparts. A finding supported by recent experimental and facial reactivity research in psychology, where gender differences in the expression of emotions during social interactions (*expresser* side) have highlighted a female susceptibility to emotional expressions (Wiggert et al., 2015). It is also interesting to note that women also rate themselves as emotionally more expressive than males (Simon & Nath, 2004). One of the reasons articulated more broadly within the psychology literature regarding this increased contagency for females relates to greater emotional awareness, often referred to as emotional intelligence (Sánchez-Núñez et al., 2008); with women reported to pay more attention to the emotions of others, which in turn increased their emotional susceptibility (Hatfield et al., 2014). It has also been reported that athlete leaders in female sports teams are more emotionally expressive (Tamminen & Bennett, 2017). Further challenges were highlighted by participants in terms of the transfer of expectations and approaches from male rugby into the women’s game. This view is consistent with the view that rugby as a sport is dominated by men and valued forms of masculinities (Ryan & Dickson, 2018), where a masculine hegemony still persists where masculinity and its attributes are lauded, with femininity attributes view less positively (Naidu-Young et al., 2024). While women’s teams exist, often this is within clubs dominated by men in senior roles, leading to often extremely engendered organizations, naturalizing male dominance in core roles (Acker, 2006; Bryan et al., 2021). Suggesting a discourse of masculine superiority over women (Bowes & Culvin, 2021; O’Brien et al., 2025), a perspective highlighted in the current study and the application of masculine leadership expectations on the women in this study.

Having empathy for team members was suggested by participants to be an important part of the role. Empathy in this context can be described to be “the capacity to share the feelings of others” (Singer & Klimecki, 2014, p. R875) and additionally as the capacity to place oneself in another’s position and understand their peculiar inner world precisely stating their feelings and thoughts (Dökmen, 2005). Empathy is important for leadership positions as it has been suggested to play a crucial and effective role in ensuring communication and mutual trust between the leader and the followers (Dubrin, 2001). It has also been suggested that empathic leadership can improve the quality of communication and interactions within a team (Dubrin, 2001). Crucial to the ability to have empathy as a leader is the process of decentering, which has been described as “the act of imagining what is significant from another person’s perspective” (Main et al., 2017, p. 358). Empathy has been suggested to be a skill rather than an innate ability, with the implication that the ability to show empathy can be developed through deliberate effort and practice (Lanzoni, 2018). Interestingly, women have been reported to both be more likely to display empathy as leaders and look for empathy in their leaders (Cundiff & Komarraju, 2008).

The importance of shared leadership within the team was a strong theme that emerged in the current study. There has been an increasing focus on shared leadership within sports teams in recent years (e.g., Fransen et al., 2015; Lee et al., 2024; Mertens et al., 2021a) with several studies reporting that as teams evolve towards shared leadership both team functioning and performance improve (Mertens et al., 2021a). Indeed, adopting a shared leadership approach has been reported to be a more effective leadership structure than having a single leader (captain) (Leo et al., 2019). Interestingly it has also been reported that the preferred nature of this shared leadership differs between male and female teams (Leo et al., 2019).

The implications of this research for practice are clear, a greater focus is required on the leadership needs of female sports teams as a distinct and different consideration to male sports teams. While there any many similarities in the leadership needs of the teams, and as a result to the demands of the captaincy role, there are also important differences. The leadership needs of female teams should be considered in the selection and training/development of female team leaders.

## 5. Conclusions

The aim of this study was to better understand the nature of leadership roles within women’s rugby at the elite level. Seeking to understand the perceptions and experiences of captains in exploring the role of the captain. One significant outcome to emerge from this study was the differences in female leadership approach and team leadership when compared to existing research on male teams. As such more research is required that seeks to understand the leadership needs of female sports teams, and the associated team leadership needs. Empathy, empowerment and collaboration appear to be key hallmarks of leadership within the domain of elite women’s rugby union, factors that need to receive greater consideration. In addition, team leaders and coaches need to consider how these key characteristics can be nurtured and developed. In addition, it is crucial to ensure that masculinity-based views of the game, and how leadership functions should not be automatically applied to the women’s game. Which can be a challenge in male dominated rugby clubs, particularly in senior leadership positions. Finally, the information presented in this research can be further utilized to better inform future leadership development programmes and captaincy selection in women’s rugby, that are based upon the needs of the players, and the team in women’s rugby.

## Figures and Tables

**Table 1:** Emerging super and sub-ordinate themes relating to participant leadership experience.

1. Factors influencing success	Authentic leadership Link with the coach Being a starter Relationships Being human Sharing leadership
2. Challenges	Anxiety inducing Lonely at the top Negative impact on behaviour Difficult players Permanence of the role Thrown in at the deep end Lack of role clarity Transitioning into the role
3. Leader characteristics	Be yourself Empathy Flexible leadership Personalising your approach Inclusive
4. Role models	Past captains Being hard as nails Bad role models Nurturing
5. Aspects of the role	Link to the coach Motivational role On pitch decision making Peacemaker Doing the washing Providing energy Importance of being a starter Selection Social leadership Relationship building
6. Types of captains	Abrasive leaders Nurturing Differentiated by level Old school
7. Leading by example	Practice/training In games Socially
8. Selection	Asking to be captain Captain at all levels Who to select Playing position
9. The women’s game	Gender inequalities Perceptions of the leader Community Context of the game
10. Advice to new captains	leadership is a journey Develop your own leadership style Lean on others in the team

## Data Availability

The datasets presented in this article are not readily available because of the personal nature of the data and the fact that participants could be identified by the full data set. Requests to access the datasets should be directed to stewart.cotterill@hsu.ac.uk.
